# Geopropolis from *Melipona orbignyi* and *Melipona quadrifasciata anthidioides* Enhances Oxidative Stress Resistance and Lifespan in *Caenorhabditis elegans*

**DOI:** 10.3390/ph19030433

**Published:** 2026-03-06

**Authors:** Helder Freitas dos Santos, Jaqueline Ferreira Campos, José Benedito Perrella Balestieri, Daniel Ferreira Leite, Alex Santos Oliveira, Wellington Henrique Botelho, Paola dos Santos da Rocha, Debora da Silva Baldivia, Sikiru Olaitan Balogun, Kely de Picoli Souza, Edson Lucas dos Santos

**Affiliations:** 1 Research Group on Biotechnology and Bioprospecting Applied to Metabolism and Cancer (GEBBAM), Universidade Federal da Grande Dourados, Dourados-Itahum Highway, Km 12, Dourados 79804-970, MS, Brazil; helderspk@gmail.com (H.F.d.S.); jaquelinefcampos@ufgd.edu.br (J.F.C.); danielleitesci@gmail.com (D.F.L.); alexsantosoliveira@gmail.com (A.S.O.); wellington.botelho365@academico.ufgd.edu.br (W.H.B.); paolarocha.biologa@gmail.com (P.d.S.d.R.); debora.s.baldivia@gmail.com (D.d.S.B.); balogun.zhikrullah@gmail.com (S.O.B.); kelypicoli@gmail.com (K.d.P.S.); 2 Programa de Pós-Graduação em Ciências da Saúde, Faculdade de Ciências da Saúde, Universidade Federal da Grande Dourados, Dourados 79804-970, MS, Brazil; 3 Faculdade do Vale do Juruena (Faculdade AJES), Avenida Gabriel Muller 1086 N, Modulo 01, Juína 78320-000, MT, Brazil

**Keywords:** bee product, biotechnological product, protective, oxidative stress, lifespan, geopropolis, *Caenorhabditis elegans*

## Abstract

**Background:** Oxidative stress arises from an imbalance in redox homeostasis, leading to the accumulation of reactive oxygen species. This condition is associated with premature aging, as well as the progression of several chronic noncommunicable diseases. Among the natural products, geopropolis stands out as a source of molecules with different biological properties. Despite reports of its therapeutic potential, data on the effects on biomolecules and lifespan remains unexplored. **Objectives:** In this context, we investigated the effects of hydroethanolic geopropolis extracts of *Melipona orbignyi* and *Melipona quadrifasciata anthidioides* on in vitro and in vivo protection against oxidative stress, as well as their toxicity and effects on lifespan. **Methods:** Firstly, we assessed the effect on protein integrity under AAPH-induced oxidative stress and on DNA stability following exposure to hydrogen peroxide and UV radiation. Furthermore, we evaluated the extracts toxicity, protection against juglone-induced oxidative stress and thermal stress, and effects on longevity in a *Caenorhabditis elegans* preclinical model. **Results:** In vitro, both extracts protected bovine serum albumin (BSA) from AAPH-induced oxidation, with maximum BSA integrity reaching 98.2 ± 1.8% (HGMO) and 91.7 ± 3.0% (HGMQ). In a UV/H_2_O_2_ plasmid assay, both extracts protected against oxidative DNA fragmentation across the tested range, achieving 100% protection (fully preserved DNA integrity) at the highest evaluated concentrations. In vivo, HGMO and HGMQ showed no acute toxicity (24–48 h), with survival comparable to controls, and increased survival under juglone-induced oxidative stress (80 µM, 24 h), with maximum viability gains of 37.3% (HGMO) and 23.9% (HGMQ). Both extracts extended lifespan, increasing maximum lifespan from 24 to 32 days (+33%). **Conclusions**: Overall, these findings support geopropolis extracts as promising candidates for biotechnological products targeting oxidative stress and healthy aging.

## 1. Introduction

Reactive oxygen species (ROS) are highly reactive molecules produced naturally as byproducts of cellular metabolism. At physiological levels, ROS act as beneficial signaling molecules that regulate cellular processes; however, as their concentration increases, they may trigger adaptive responses and ultimately exert deleterious effects [1]. The balance between the generation and neutralization of ROS in the organism is responsible for ensuring cellular redox homeostasis [2]. However, when the production of ROS exceeds the capacity of the endogenous antioxidant defense system, a redox imbalance occurs, triggering oxidative stress.

Oxidative stress is defined by elevated levels of ROS in the body, loss of redox homeostasis, and cytotoxic effects on cellular and tissue function [3,4]. At the molecular level, oxidative stress causes damage to lipids, proteins, and nucleic acids, events associated with the acceleration of cellular aging and the onset of chronic noncommunicable diseases [5].

Chronic noncommunicable diseases include cardiovascular, respiratory, diabetes, neurodegenerative, and cancer diseases, which together account for approximately 74% of all deaths worldwide [6]. There is a consensus that oxidative stress is associated with the onset, progression, or worsening of these diseases [7]. During aging, ROS generates cumulative and irreversible damage to cells, affecting proper cellular functioning and increasing functional deficits in the body [8,9]. From this perspective, obtaining antioxidant molecules of exogenous origin is essential for health, contributing to the reduction in the harmful effects of oxidative stress and the prevention of diseases related to this condition.

Natural products are an immeasurable source of chemical compounds that can bring benefits to human health, particularly due to their antioxidant action, capable of neutralizing the harmful effects of ROS and preventing diseases [2]. Among natural products, geopropolis is a substance uniquely produced by certain species of stingless bees [10] from diverse natural materials, including plant exudates, pollen, wax, mandibular secretions, and soil [11].

This bee product has gained prominence in recent years, where its emerging biological potential has been demonstrated through its antimicrobial [12], anti-inflammatory [13], antiviral [14], wound healing [15], anti-leishmaniasis [16], gastroprotective [17], anticancer [18], and antioxidant [19,20] properties. In previous studies, we demonstrated the antimicrobial, anti-inflammatory, and antimutagenic properties, as well as a potent antioxidant action against free radicals and lipid peroxidation, of geopropolis of the species *M. orbignyi* and *M. q. anthidioides* [12,19]. However, there is a lack of studies assessing the protective action on macromolecules in vitro and their antioxidant effects in vivo.

The aim and objectives of the present study were to investigate the protective effect of the hydroethanolic extracts of geopropolis from *M. orbignyi* (HGMO) and *M. q. anthidioides* (HGMQ) against oxidative damage to proteins and DNA and the potential toxicity and protection against oxidative and thermal stress, as well as their influence on the modulation of *Caenorhabditis elegans* lifespan.

## 2. Results

### 2.1. Protein Oxidation

**Figure 1:**
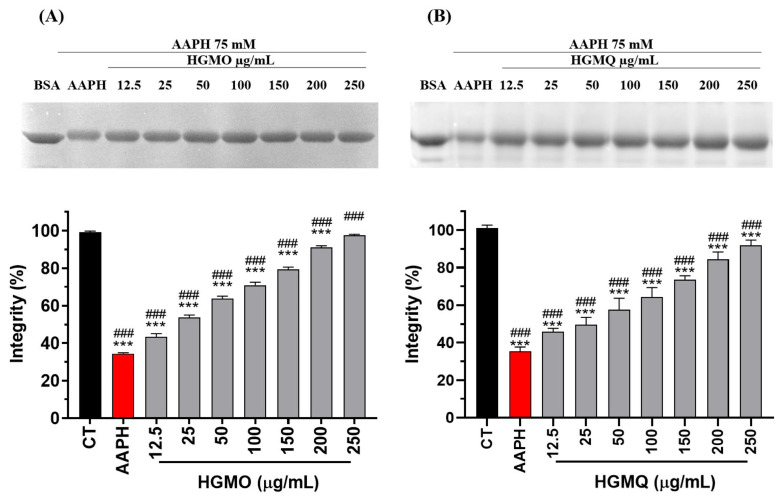
Protection against AAPH-induced protein oxidation (75 mM) promoted by HGMO (**A**) and HGMQ (**B**) at different concentrations. Values are expressed as mean ± SEM. * vs. CT; # vs. AAPH. ***, ### *p* < 0.001. AAPH: 2,2′-Azobis(2-amidinopropane) dihydrochloride BSA: bovine serum albumin; HGMO and HGMQ geopropolis hydroethanolic extracts of *Melipona orbignyi* and *Melipona quadrifasciata anthidioides* respectively. Red bars indicate the positive control corresponding to oxidative damage conditions.

The assay assesses the protective effect of the extracts against AAPH-induced oxidative damage to BSA, with untreated BSA serving as a negative control and AAPH-treated BSA as a positive control. The control AAPH reduced BSA integrity to about 35% when compared to the negative control. In contrast, both HGMO and HGMQ extracts exhibited significantly protective effects by reducing BSA oxidation at all tested concentrations, maintaining BSA integrity with values comparable to the negative control at the highest concentrations. Figure 1A,B shows a concentration-dependent increase in band intensity. HGMO was able to maintain BSA integrity at 43.7 ± 5.1 and 98.2 ± 1.8% at concentrations of 12.5 and 250 µg/mL, respectively (Figure 1A). HGMQ maintained BSA integrity at 45.6 ± 2.0 and 91.7 ± 3.0% at the same concentrations, respectively (Figure 1B).

### 2.2. DNA-Induced Oxidative Damage

**Figure 2:**
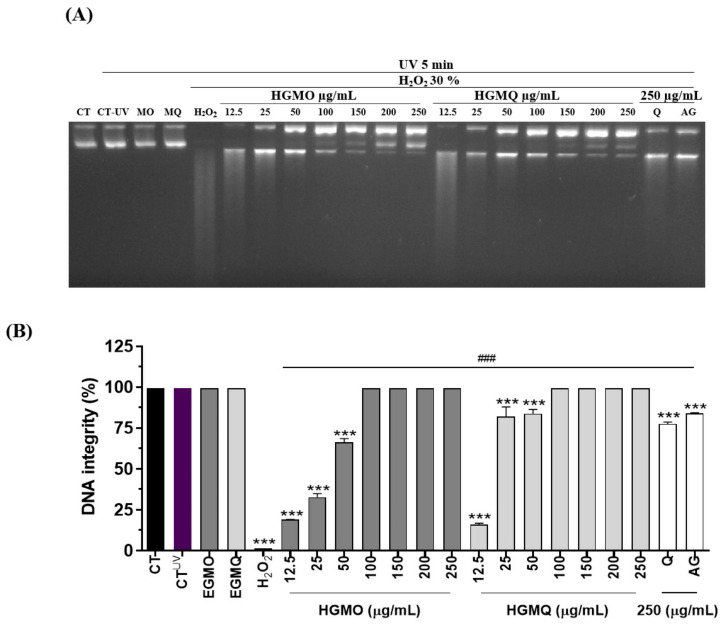
Depicts the protection against oxidative fragmentation in plasmid DNA induced by UV radiation and H_2_O_2_ (30%) promoted by HGMO/HGMQ at different concentrations. (**A**) Representative image of agarose gel with the respective treatments. (**B**) DNA fragmentation (%). Values are expressed as mean ± SEM. # vs. CT; * vs. UV/H_2_O_2_; ***, ### *p* < 0.001. CT: untreated control; CT-UV: plasmid DNA exposed to UV radiation; H_2_O_2_: plasmid DNA exposed to hydrogen peroxide (30%); Q: quercetin (positive antioxidant control); AG: gallic acid (positive antioxidant control). HGMO and HGMQ geopropolis hydroethanolic extracts of *Melipona orbignyi* and *Melipona quadrifasciata anthidioides* respectively. Purple bar indicates the positive control corresponding to oxidative damage conditions.

The extracts were assessed for their ability to prevent plasmid DNA fragmentation caused by ultraviolet radiation and hydrogen peroxide (H_2_O_2_). As a result, we demonstrated that both geopropolis extracts, HGMO and HGMQ, presented significantly protective action against oxidative fragmentation of nucleic acid, as shown in Figure 2A,B.

In the positive control exposed to UV radiation and H_2_O_2_, a total loss of DNA integrity was observed. However, in the presence of the extracts, DNA integrity was protected in a concentration-dependent manner, showing higher integrity than samples incubated with the control antioxidants quercetin and gallic acid, under the tested conditions. At the lowest concentrations, up to 50 µg/mL, HGMO preserved DNA integrity at levels of 18.5 ± 0.07, 32.6 ± 3.04, and 66.3 ± 3.11%. HGMQ maintained DNA integrity at levels of 15.8 ± 1.27, 81.9 ± 8.41, and 83.8 ± 3.46%. On the other hand, at the highest concentrations (100–250 µg/mL) of both extracts, DNA integrity was markedly preserved under these experimental conditions, corresponding to 100% DNA integrity in this assay.

### 2.3. In Vivo Tests Using the Caenorhabditis elegans Model

#### 2.3.1. Toxicity

**Figure 3:**
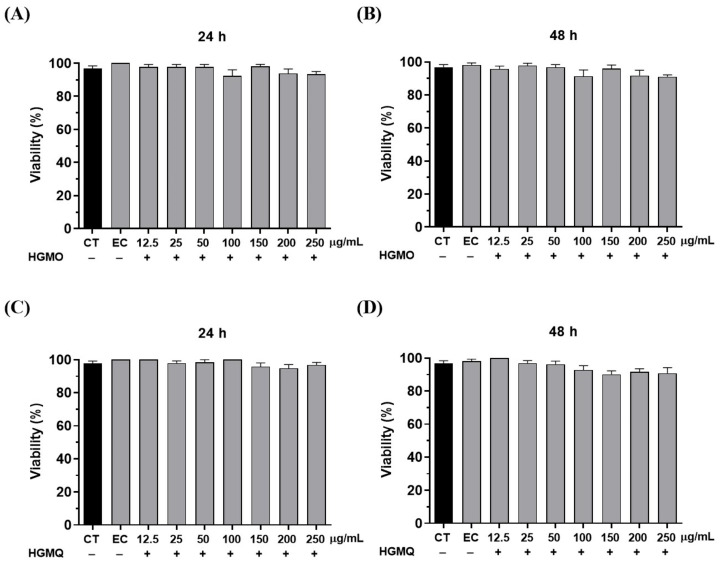
Toxicity of geopropolis extracts on *C. elegans* in the periods of (**A**) 24 h and (**B**) 48 h for HGMO and (**C**) 24 h and (**D**) 48 h for HGMQ. Values are expressed as mean ± SEM. CT: negative control. EC: ethanol control. HGMO and HGMQ geopropolis hydroethanolic extracts of *Melipona orbignyi* and *Melipona quadrifasciata anthidioides* respectively.

In the in vivo studies, the first parameter evaluated was toxicity. In this test, the nematodes were treated with HGMO or HGMQ. As shown in Figure 3A–D, after 24 and 48 h, no toxic effects were observed in any of the tested concentrations (ranging from 12.5 to 250 µg/mL). Once the safe dosage for use was determined, subsequent tests were conducted based on the observed responses.

#### 2.3.2. Protection Against Oxidative Stress Induced by Juglone

**Figure 4:**
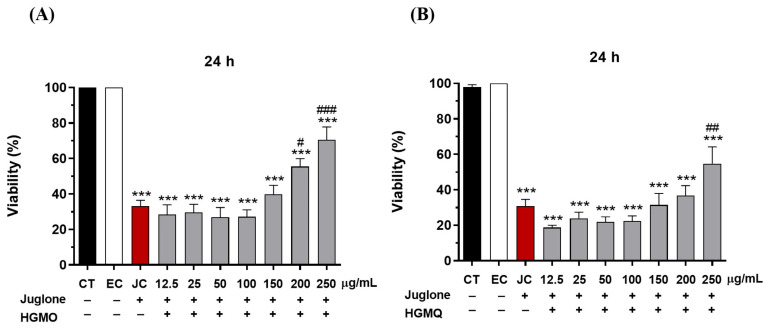
Protective effects of HGMO (**A**) and HGMQ (**B**) against oxidative stress induced by juglone after 24 h in *C. elegans*. EC: ethanol control, JC: juglone control. Values are expressed as mean ± SEM. * vs. CT; # vs. JC; *p* < 0.05; ## *p* < 0.01; ***, ### *p* < 0.001. HGMO and HGMQ geopropolis hydroethanolic extracts of *Melipona orbignyi* and *Melipona quadrifasciata anthidioides* respectively. Red bars indicate the positive control corresponding to oxidative damage conditions.

The protective effects of HGMO and HGMQ against oxidative stress were evaluated by assessing the survival of *C. elegans* exposed to Juglone (80 µM) over 24 h. As shown in Figure 4A,B, the oxidizing agent reduced the viability of the control group by approximately 70% (33.2 ± 3.2% of viability). In contrast, nematodes treated with concentrations of 200 µg/mL and 250 µg/mL of HGMO exhibited significant increases in survival of 22.3% (55.5 ± 4.3% of viability) and 37.3% (70.5 ± 7.3% of viability), respectively. Additionally, nematodes treated with 250 µg/mL of HGMQ showed a 23.9% (54.8 ± 9.4 of viability) increase in survival, as illustrated in Figure 4A,B.

#### 2.3.3. Protection Against Thermal Stress

**Figure 5:**
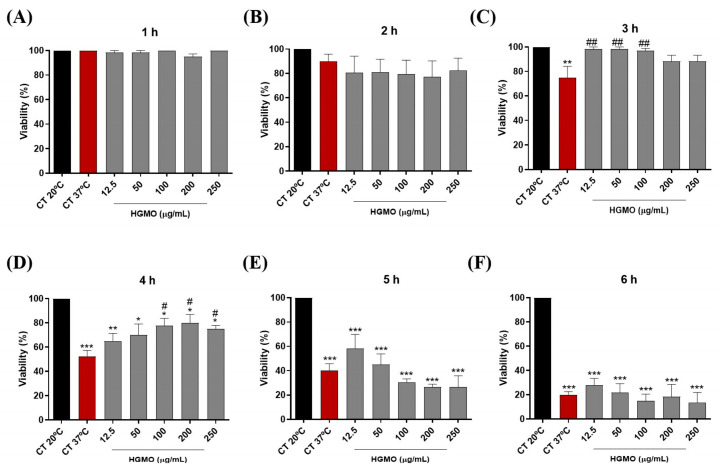
Effects of HGMO on *C. elegans* viability after 1 (**A**), 2 (**B**), 3 (**C**), 4 (**D**), 5 (**E**), and 6 h (**F**) of heat stress induction. Values are expressed as mean ± SEM. * vs. CT 20 °C; # vs. CT 37 °C; *, # *p* < 0.05; **, ## *p* < 0.01; *** *p* < 0.001. HGMO and HGMQ geopropolis hydroethanolic extracts of *Melipona orbignyi* and *Melipona quadrifasciata anthidioides* respectively. Red bars indicate the positive control corresponding to thermal stress conditions.

**Figure 6:**
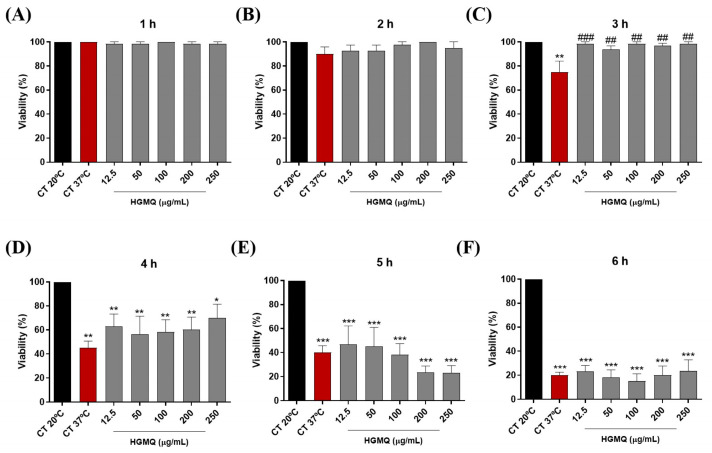
Effects of HGMQ on *C. elegans* viability after 1 (**A**), 2 (**B**), 3 (**C**), 4 (**D**), 5 (**E**), and 6 h (**F**) of heat stress induction. Values are expressed as mean ± SEM. * vs. CT 20 °C; # vs. CT 37 °C; *, *p* < 0.05; **, ## *p* < 0.01; ***, ### *p* < 0.001. HGMO and HGMQ geopropolis hydroethanolic extracts of *Melipona orbignyi* and *Melipona quadrifasciata anthidioides* respectively. Red bars indicate the positive control corresponding to thermal stress conditions.

In the heat stress assay, the increase in temperature led to a progressive reduction in the viability of control nematodes, reaching a reduction of approximately 80% after 6 h at 37 °C (Figure 5A–F and Figure 6A–F). However, the results show that both geopropolis extracts exhibited protective action against heat stress. As shown in Figure 5C, the positive control (37 °C) resulted in approximately 75% nematode viability, whereas HGMO (12.5–100 µg/mL) maintained viability at ~98.3%, similar to the negative control (20 °C), over the 3 h period. Moreover, this extract also demonstrated protection over the 4-h period at concentrations of 100, 200, and 250 µg/mL, maintaining nematode viability between approximately 75–80%, compared with 52.5% viability observed in the positive control (37 °C) (Figure 5D). In Figure 6C, HGMQ maintained nematode viability over the 3-h period at all concentrations evaluated. While the positive control (37 °C) showed approximately 75% viability, all tested concentrations showed viability levels similar to the negative control.

#### 2.3.4. Longevity Assay

**Figure 7:**
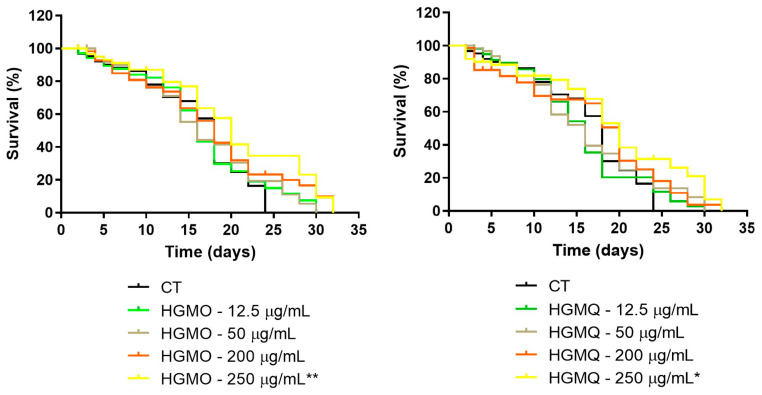
Effects of geopropolis extracts on *C. elegans* lifespan. (**A**) Nematodes treated with different concentrations of HGMO and (**B**) HGMQ. * Represents statistically significant results (* *p* < 0.05 and ** *p* < 0.01) when the treated group was compared with the control group. Values are expressed as mean ± SEM. HGMO and HGMQ geopropolis hydroethanolic extracts of *Melipona orbignyi* and *Melipona quadrifasciata anthidioides* respectively.

The effects of HGMO and HGMQ on the lifespan of C. elegans are summarized in Table 1 and illustrated by the survival curves in Figure 7A,B. Both extracts promoted an extension of nematode lifespan, with statistically significant effects on mean lifespan observed at the highest concentration tested (250 µg/mL). Analysis of the survival curves revealed that treatment with HGMO at concentrations of 12.5 and 50 µg/mL extended the maximum lifespan from 24 days in the control group to 30 days (25% increase), whereas treatment with 200 and 250 µg/mL prolonged the maximum lifespan to 32 days (33% increase) (Figure 7A). Nematodes treated with HGMQ exhibited a comparable pattern of maximum lifespan extension across the evaluated concentrations (Figure 7B).

Additionally, the highest concentration of both extracts was able to increase the mean lifespan of *C. elegans* by approximately 11%, when compared to the control group, as shown in Table 1.

## 3. Discussion

Natural products have a unique structural diversity of chemical components, which can confer different biological activities [21]. Among these, geopropolis is a product of beekeeping origin, produced and used by stingless bees in their nests as a construction material, as it is a mixture of propolis and soil [10]. Geopropolis has been reported in ethnomedicinal practices for the treatment of infectious, respiratory, and ocular conditions [22]. Despite its use for medicinal purposes, studies about geopropolis are still scarce, especially on its pharmacological properties.

Previous studies from our group showed that geopropolis produced by the stingless bees *Melipona orbignyi* and *Melipona quadrifasciata anthidioides* exhibits antimicrobial, anti-inflammatory, antimutagenic, and antioxidant activities [12,19]. We also reported the chemical characterization of both hydroalcoholic extracts [12,19]. A summary of the major compounds identified in each extract is provided in the Supplementary Materials (Table S1) to facilitate comparison. Building on these findings, and given that the pharmacological properties of this bee product remain incompletely characterized, we show here that HGMO and HGMQ attenuate AAPH-induced protein oxidative damage.

Proteins are macromolecules that are fundamental to cell biology and are very sensitive to oxidative damage, which can alter their structure and cause loss of function [23]. Oxidative damage to proteins alters the activity of enzymes and receptors, signal transduction mechanisms, and susceptibility to proteolysis [24]. ROS promotes damage to different amino acids and triggers oxidative structural changes in proteins, such as fragmentation and/or aggregate formation [25,26]. Thus, the deleterious effects resulting from oxidative stress are significant factors in neurodegenerative diseases [27].

In addition to the protective effect on proteins, HGMO and HGMQ were also able to protect DNA against oxidative damage caused by hydroxyl radicals, generated through the photolysis of H_2_O_2_ induced by UV light [28]. Although this model employs non-physiological oxidative conditions, it is widely used as a stringent in vitro system to indicate direct chemical protection of isolated DNA under severe oxidative challenge.

DNA is a macromolecule extremely sensitive to oxidative damage. Hydroxyl radicals react with DNA bases, abstracting electrons or hydrogen atoms, and promoting the cleavage and breakage of the DNA strand [29,30]. Oxidative damage to DNA can accumulate within its structure and trigger genomic instability, increasing the likelihood of mutations, a scenario related to the decline of cellular function, aging, and the carcinogenic process [31,32]. The extracts also had a more efficient protective action compared to the standard controls of gallic acid and quercetin. This effect may be due to the synergism between the bioactive molecules present in the extracts.

It is possible to find a diverse presence of chemical components in geopropolis from different stingless bees, such as phenolic acids (gallic, caffeic, syringic, benzoic) [33], flavonoids as vanillic acid, rutin, aromadendrin, quercetin [34] naringerin [14], ferreirin and dihydrokaempferide [18], hydrolyzable tannins (gallotannins and ellagitannins) [16], alkaloids [35], and terpenes [18,19]. The literature reports that phenolic compounds such as flavonoids exhibit antioxidant activity through different mechanisms, such as: (I) donating electrons and hydrogen atoms to stabilize free radicals, (II) chelating ROS-catalyzing metal ions, and (III) regulating oxidases and antioxidant enzymes [36]. In addition, some studies have reported the ability of certain molecules to bind to DNA and prevent oxidative damage [37].

From this perspective, the protection promoted by the extracts on proteins and DNA is possibly related to the presence of bioactive substances previously identified in the geopropolis of *M. orbignyi* and *M. q. anthidioides*, such as flavonoids naringin, aromadendrin, and methyl aromadendrin [12,19]. Additionally, these previous studies demonstrated that one of the antioxidant mechanisms of HGMO/HGMQ is the donation of electrons for the stabilization of free radicals, whether hydrophilic or lipophilic [12,19].

Comparative analysis of these previously reported chemical profiles indicates that, although HGMO and HGMQ share several phenolic and flavonoid constituents, HGMO exhibited greater chemical diversity (26 identified compounds versus 20 in HGMQ) [12,19], including additional terpenoid derivatives and methylated flavonoids such as methyl naringenin. These compositional differences may influence antioxidant efficiency and biological responses, since redox potential and bioactivity depend on structural diversity and potential synergistic interactions among constituents.

After confirming the protective effects of the extracts on biomolecules, we proceeded to evaluate their activity in vivo using the nematode *C. elegans*. This organism is genetically well characterized [38] and is widely used as a preclinical toxicity model due to its strong correlation with mammalian toxicity outcomes [39] as well as the presence of numerous genes homologous to those in humans [40]. Geopropolis extracts did not induce toxicity at any of the concentrations evaluated in *C. elegans*. Absence of toxicity was also observed in the propolis extract (2–8 mg/mL) of the species *Heterotrigona itama* in *C. elegans* [41], as well as for propolis of *Plebeia catamarcensis* and *Tetragonisca fiebrigi*, which showed no toxicity up to concentrations of 1500 µg/mL [42]. Additionally, no toxicity was detected for the coumarin isolated from the geopropolis of *Melipona scutellaris*, Cinnamoyloxy-mammeisin (2–20 mg/kg), in the *Galleria mellonella* model [43].

In the absence of toxicity, the pharmacological potential of the extracts becomes even more relevant. Regarding protection against oxidative stress, the extracts increased the viability of *C. elegans* exposed to juglone. The selection of juglone as the stressor was strategic, as it provides insights that go beyond simple antioxidant capacity. Juglone, a pro-oxidant, generates a continuous flux of intracellular superoxide radicals via a well-characterized redox cycling mechanism, thus mimicking an endogenous oxidative challenge [44]. Crucially, survival against juglone-induced toxicity in *C. elegans* is functionally dependent on the activation of the master transcription factors DAF-16/FOXO and SKN-1/Nrf2, which are central regulators of longevity and cellular stress resistance [45].

In this sense, an important pathway related to the response to oxidative stress (homologous in mammals) is the insulin/IGF-1 pathway [46]. This pathway is initiated by the daf-2 receptor (IGFR in humans), which negatively regulates the transcriptional factors *daf-16* and *Skn-1*, responsible for the expression of genes associated with longevity and transcriptional control of antioxidant enzymes [40,41,47]. *Daf-16* and *Skn-1* are functional orthologs of the transcriptional factors *FoxO-1* and *Nrf2* in mammals [48]. In the literature, it is reported that phenolic compounds present in propolis and other natural products are capable of positively modulating *daf-16* and *Snk-1* [49]. Therefore, the increased survival observed in our study strongly suggests that the protective effects of geopropolis are not merely due to direct ROS scavenging, but are likely mediated by the potentiation of the nematode’s intrinsic, genetically programmed defense pathways. This established model provides a physiologically relevant assessment, indicating that the bioactive compounds within geopropolis may booster these conserved cellular defense mechanisms, a finding of significant interest for promoting healthspan.

In addition to chemical induction, heat stress is a condition that can induce oxidative stress by increasing the temperature to which the animal is subjected [50]. During this type of stress, *C. elegans* cells activate the heat shock response mechanism regulated by the transcription factor *HSF-1* (heat shock factor-1), which in turn regulates the expression levels of specific defense proteins, known as chaperones or heat shock proteins (HSPs) [51,52]. Animals treated with both extracts exhibited increased viability under heat stress, indicating enhanced thermotolerance.

It is worth noting that the protection of the extracts occurred only after 3 h or after 3 and 4 h, which suggests that after a certain period of heat stress, the extracts are unable to improve the recovery of the animals due to the severity of the damage generated during the last hour of stress. In the study by Jovic et al. [53], exposure to heat stress for more than 4 h irreversibly compromised the survival, reproduction, and movement of the animals.

During heat stress, proteins can become misfolded, altering their conformation and potentially leading to the formation of protein aggregates [38,52].

Several signaling pathways have been reported to participate in cellular responses to different stressors, such as inflammatory cytokines, UV radiation, oxidative stress, and heat stress, including the c-Jun N-terminal kinase (JNK) signaling pathway [54]. According to previous studies, upon detecting stressful stimuli, the JNK-1 protein is able to activate *daf-16* and promote its translocation to the nucleus, where it will induce the expression of target genes responsible for avoiding harmful stresses [47,54]. Heat stress is able to drive the translocation of *daf-16* to the nucleus [55], which in turn activates the heat shock response, promoting adaptation and survival in nematodes [52].

After demonstrating that geopropolis extracts increased the survival of *C. elegans* under abiotic stress (oxidative and thermal), we examined the effects on the longevity of nematodes treated daily with HGMO and HGMQ until all were dead.

Based on survival curve analysis, both extracts were able to extend the lifespan of the nematodes at all evaluated concentrations.

This study is the first to evaluate the effects of geopropolis on the *C. elegans* model, including its impact on longevity. This effect on lifespan may be attributed to the antioxidant properties of geopropolis extracts throughout their life. Additionally, this effect may be associated, based on the literature evidence, with pathways involving transcription factors such as *daf-16* and *skn-1*, which are widely recognized as key regulators of oxidative stress resistance and longevity in *C. elegans*.

The flavonoid naringenin (present in our extracts) was investigated by Ge et al. [56], finding an increase in lifespan in *C. elegans* under normal and oxidative stress conditions. In this study, naringenin improved locomotion, delayed paralysis, reversed defective chemotaxis induced by beta-amyloid protein, increased the activity of antioxidant enzymes, and reduced ROS and MDA content. Furthermore, it positively modulated the transcription factors *daf-16* and *Skn-1* while negatively modulating daf-2, indicating that it promotes longevity through the insulin/IGF-1 pathway and MAPK signaling pathways.

The insulin/IGF-1 receptor and JNK (MAPK) signaling pathways are important signaling cascades involved in cellular responses to stress [57]. According to the established models, positive signaling of the daf-2 pathway inhibits the activity of *daf-16* and *Skn-1*, while positive signaling of the JNK pathway leads to the activation of *daf-16*. Once activated, *daf-16* induces the expression of antioxidant enzyme genes and heat shock proteins, promoting increased stress resistance and longevity [38,41,47].

## 4. Materials and Methods

### 4.1. Chemicals and Reagents

All reagents used in the experiments were of analytical grade and purchased from Sigma-Aldrich/Merck (São Paulo, Brazil).

### 4.2. Geopropolis Sample Collection and Preparation of Hydroethanolic Extracts

Samples of geopropolis produced by the stingless bees *Melipona orbignyi* and *Melipona quadrifasciata anthidioides* (SISGEN Registration No. AF5168A) were identified and collected by biologist Prof. Dr. José Benedito Perrela Balestieri at a meliponary in Dourados, Mato Grosso do Sul state, Brazil (22°13′12″ S and 54°49′2″ W). The samples consist of a heterogeneous matrix composed of soil-like fragments mixed with propolis material. After collection, the raw material was stored at −20 °C until extraction. Crucially, the specific hydroethanolic extracts evaluated in the present study (HGMO and HGMQ) are the identical batches previously prepared and comprehensively chemically characterized by our research group in our previous studies [12,19]. Consequently, the chemical composition data detailed in these prior works directly apply to the bioassays performed herein.

Briefly, the extraction procedure followed the previously validated methodology. The geopropolis samples were macerated using a mortar and pestle and homogenized with 70% ethanol at a ratio of 1:3 (*w*/*v*). The resulting solutions were incubated in appropriate glass flasks in a shaker under moderate agitation (175 rpm), in the dark, at room temperature for 24 h. Subsequently, the solutions were filtered through 80 g/m^2^ qualitative filter paper (ProLab, São Paulo, Brazil), concentrated using a rotary vacuum evaporator at 40 °C (Gehaka, São Paulo, Brazil), and lyophilized to yield the final hydroethanolic extracts. The extraction yields were 14.9% for *M. orbignyi* (HGMO) and 6.0% for *M. q. anthidioides* (HGMQ), calculated using the formula:

Extraction yield (%) = (lyophilized sample × 100)/(original sample)

The extracts obtained were kept at −20 °C and protected from light until the analyses were performed.

### 4.3. Macromolecule Protection Assays

#### 4.3.1. Protein Oxidation

The effect of the extracts on protein oxidation was evaluated using an oxidative induction assay with bovine serum albumin (BSA) and AAPH (2,2′-azobis(2-amidinopropane) dihydrochloride) as the oxidizing agent. In microtubes, 15 µL of BSA (1.5 mg/mL) was incubated together with 15 µL of HGMO or HGMQ, at different concentrations (12.5–250 µg/mL) for 30 min. Subsequently, 15 µL of the AAPH solution (75 mM) was added to each microtube, followed by incubation for 120 min at 37 °C. The samples were then mixed with 75 µL of sample buffer (40% glycerol, 8% SDS, 0.25 mM Tris-HCl pH 6.8; 20% β-mercaptoethanol, 0.01% bromophenol blue) and heated at 100 °C for 3 min. After this, the samples were applied to agarose gels and subjected to electrophoresis (SDS-PAGE 12%) using the Mini-PROTEAN Tetra Cell system (Bio-Rad Laboratories, Hercules, CA, USA) at 200 V for 60 min. The assay was performed in two independent experiments, each conducted in quadruplicate (four gels per experiment). The gels were scanned using a Gel Doc EZ Imager photodocumentation system (Bio-Rad Laboratories). Signal intensity was quantified in quadruplicate for each experiment using Image Lab software version 6.0.1 (Bio-Rad Laboratories), and the resulting values were normalized for statistical analysis.

#### 4.3.2. DNA-Induced Oxidative Damage

The effect of HGMO and HGMQ against DNA-induced oxidative damage was investigated by hydrogen peroxide (H_2_O_2_) and ultraviolet (UV) light-induced oxidative fragmentation. For this, plasmid DNA (pcDNA 3.1) (50 ng/µL) in 1x PBS solution was incubated with different concentrations of HGMO or HGMQ (12.5–250 µg/mL) and 30% H_2_O_2_ for 30 min. Then, the samples were incubated in a UVT-312 transilluminator (São Paulo, Brazil) at 302 nm, at room temperature for 5 min, followed by application and electrophoresis in 2% agarose gel containing ethidium bromide (10 ng/mL). As controls, plasmid DNA samples were also incubated with the standard antioxidants quercetin (Q) and gallic acid (GA) (250 µg/mL) and subjected to the same treatment. Subsequently, the gel was scanned in a Gel Doc EZ Imager (Bio-Rad Laboratories), and the analysis was performed using the Image Lab software. The average of the duplicates of two independent experiments was performed to obtain the data.

### 4.4. In Vivo Assays Using the Caenorhabditis elegans Model

#### 4.4.1. Cultivation and Maintenance of *C. elegans*

To perform the in vivo assays, wild-type N2 Bristrol nematodes obtained from the Caenorhabditis Genetics Center (CGC) (Minneapolis, MN, USA), were used. The nematodes were kept in an incubator at 20 °C, cultivated in Petri dishes containing Nematode Growth Medium (NGM) and fed with *Escherichia coli* (OP50) bacteria. The bacteria used as food for the animals were inactivated with the antibiotic kanamycin (10 mM). To perform the experiments, the nematode culture was synchronized using 2% sodium hypochlorite and 5 M NaOH. The eggs resistant to alkaline lysis were transferred to new Petri dishes containing NGM and *E. coli*.

#### 4.4.2. Acute Toxicity Assessment

To assess the toxic effect of acute exposure to HGMO and HGMQ, an average of 10 nematodes, synchronized in the L4 phase, were transferred to 96-well microplates containing 100 µL of M9 buffer and 100 µL of the respective extracts at different concentrations (12.5 to 250 µg/mL). Subsequently, the nematodes were incubated at 20 °C for 24 and 48 h. As a negative control, the nematodes were incubated with 200 µL of M9 buffer only. Additionally, an ethanol control was performed using the same concentration of the solvent present in the extracts. After the incubation period, viability was assessed by touch sensitivity using a platinum wire. Three independent experiments were performed in triplicate.

#### 4.4.3. Protection Against Oxidative Stress Induced by Juglone

To evaluate the effects of HGMO and HGMQ against oxidative stress, wild-type *C. elegans* nematodes were exposed to the oxidizing agent Juglone (5-hydroxy-1,4-naphthoquinone) [58]. After synchronization, an average of 10 L4-stage nematodes were placed in wells containing 100 µL of M9 buffer. The nematodes were then pre-incubated with 100 µL of different concentrations (12.5 to 250 µg/mL) of HGMO/HGMQ for 1 h. Subsequently, the oxidative stress inducer Juglone (80 µM) was added to the wells containing the nematodes and treatments, followed by incubation at 20 °C for 24 h. Nematodes incubated only with M9 buffer (250 µL) and with juglone (200 µL of M9 buffer + 50 µL of juglone) were used as negative and positive controls for oxidative damage, respectively. After the incubation period, the viability of the nematodes was assessed by touch sensitivity using a platinum wire. Three independent experiments were performed in triplicate.

#### 4.4.4. Protection Against Thermal Stress

To evaluate the effect of heat stress, N2 nematodes were synchronized at the L4 stage and treated with HGMO or HGMQ (12.5–250 μg/mL). For this purpose, 10 nematodes were transferred to new plates containing NGM, *E. coli* OP50, and the respective treatments. Heat stress was induced by increasing the culture temperature from 20 to 37 °C for 6 h, with evaluations performed at 1 h intervals. After the incubation period, the nematodes were kept at 20 °C for 16 h. This procedure allows viable nematodes to recover and be counted by touch with the aid of a platinum wire. Three independent experiments were performed in triplicate.

#### 4.4.5. Longevity Assay

To evaluate the effect on lifespan, synchronized N2 nematodes at the L4 stage were treated with HGMO/HGMQ. On the first day of the adult phase, 10 nematodes per group were transferred to new Petri dishes containing *E. coli* OP50 with or without the presence of HGMO/HGMQ at different concentrations (12.5, 50, 200, and 250 µg/mL), which was considered the first day of the experiment. During the first six days, when the animals were in the reproductive stage, the nematodes were transferred daily to new dishes containing their respective treatments. From the sixth day onwards, the nematodes were transferred from one plate to another every 2 days, and survival was assessed by classifying the animals as dead/alive daily, until all *C. elegans* died. Animals that did not move after slight stimulation with a platinum wire were considered dead. Nematodes with internally hatched eggs or not found were excluded from the analysis. Two independent experiments were performed in triplicate.

### 4.5. Statistical Analysis

All data were expressed as the mean ± standard error of the mean (SEM). Significant differences between groups were determined using Student’s *t*-test for comparison between two groups and analysis of variance (ANOVA), followed by Dunnett’s test for comparison of three or more groups using GraphPad Prism 5 software (San Diego, CA, USA). Results were considered significant when *p* < 0.05. Survival analyses were performed using Kaplan–Meier survival curves. Differences between survival distributions were evaluated using the log-rank (Mantel–Cox) test. Hazard ratios were obtained from survival analysis to estimate the relative risk of death between treated and control groups.

## 5. Conclusions

In conclusion, this study provides the first evidence that hydroalcoholic extracts of geopropolis from the stingless bees *Melipona orbignyi* and *M. q. anthidioides* confer significant protection against oxidative stress and promote longevity in the *C. elegans* model. Our findings demonstrate that these extracts are non-toxic, enhance survival under both chemical and thermal stress, and extend the overall lifespan of the nematodes. These results highlight the considerable pharmacological potential of geopropolis as a source of bioactive compounds for promoting healthspan.

Nevertheless, this study has several limitations that should be acknowledged. Firstly, while *C. elegans* is a powerful and genetically tractable model for aging and stress research, it is an invertebrate. The physiological differences in metabolism, bioavailability, and compound distribution between nematodes and mammals mean that these findings must be interpreted as a foundational proof-of-concept. Secondly, this investigation utilized crude hydroalcoholic extracts. Although this approach confirms the bioactivity of the natural product as a whole, it does not identify the specific compound or combination of compounds responsible for the protective and pro-longevity effects.

These limitations pave the way for clear future research directions. The logical next step is to validate these findings in a mammalian context, initially using in vitro models (e.g., human cell lines under oxidative stress) before progressing to in vivo rodent models of aging or stress. This will be crucial for assessing translational potential. Furthermore, to advance our mechanistic understanding, future work should focus on bioactivity-guided fractionation of the most potent extract. This process will allow for the isolation and structural elucidation of the specific active molecules, which can then be studied individually to determine their precise molecular targets and mechanisms of action, including direct confirmation of their role in modulating the *daf-16* and *skn-1* pathways.

Ultimately, this research establishes geopropolis as a promising candidate for the development of novel health-promoting agents, and the proposed future work is essential to unlock its full therapeutic potential.

## Figures and Tables

**Table 1:** Effects of hydroethanolic extracts of geopropolis from *M. orbignyi* (EGMO) and *M. q. anthidioides* (EGMQ) on *C. elegans* N2 lifespan.

Treatment (µg/mL)	Mean Lifespan ^a^ (Days)		Maximum Lifespan ^a^ (Days)		*p* Value (Log-Rank) vs. Control ^b^		Hazard Ratio ^c^ (Treated vs. Control)		Total Number of Nematodes	
	HGMO	HGMQ	HGMO	HGMQ	HGMO	HGMQ	HGMO	HGMQ	HGMO	HGMQ
0	18	18	24	24	-	-	-	-	60 (2)	60 (2)
12.5	16	16	30	30	0.7563	0.8620	0.92	1.05	60 (2)	60 (2)
50	16	16	30	30	0.3390	0.7451	0.78	0.92	60 (2)	60 (2)
200	18	20	32	32	0.1725	0.2352	0.70	0.74	60 (2)	60 (2)
250	20	20	32	32	0.0068 **	0.0285 *	0.48	0.55	60 (2)	60 (2)

* Represents statistically significant results (* *p* < 0.05 and ** *p* < 0.01) when the treated group was compared with the control group. ^a^ Lifespan measured in days. ^b^ The comparisons were performed using the log-rank test (Mantel–Cox). ^c^ Hazard ratios were obtained from survival analysis HGMO and HGMQ geopropolis hydroethanolic extracts of *Melipona orbignyi* and *Melipona quadrifasciata anthidioides* respectively.

## Data Availability

The original contributions presented in this study are included in the article/Supplementary Material. Further inquiries can be directed to the corresponding author.

## Supplementary Materials

The following supporting information can be downloaded at https://www.mdpi.com/article/10.3390/ph19030433/s1, Table S1: The main compounds identified in the Geopropolis (HGMO vs. HGMQ) extract.
