# Supplementary material for: Geopropolis from Melipona orbignyi and Melipona quadrifasciata anthidioides Enhances Oxidative Stress Resistance and Lifespan in Caenorhabditis elegans
Source: Pharmaceuticals (Basel). 2026 Mar 6;19(3):433. doi: 10.3390/ph19030433 (PMC13028737; doi:10.3390/ph19030433)
Supplement: Supplementary file 1 [file pharmaceuticals-19-00433-s001.zip › pharmaceuticals-4124184-supplementary.pdf]

Table S1: The main compounds identified in the Geopropolis (HGMO vs. HGMQ) extracts

Compounds class	Compound name	Found in HGMO (***M. orbignyi***) [19]	Found in HGMQ (***M. q. anthidioides***) [12]
Flavonoids	Aromadendrin	Yes	Yes
	Naringenin	Yes	Yes
	Methyl Aromadendrin	Yes	Yes
	Methyl naringenin	Yes	-
Phenolic Acids & Derivatives	O-Coumaroyl O-galloyl-hexoside	Yes	Yes
	Di-O-galloyl O-coumaroyl-hexoside	Yes	Yes
	O-Cinnamoyl O-galloyl-hexoside	Yes	Yes
	Di-O-galloyl O-cinnamoyl-hexoside	Yes	Yes
	Di-O-coumaroyl-hexoside	Yes	Yes
	Di-O-coumaroyl O-galloyl-hexoside	Yes	Yes
	O-Cinnamoyl O-coumaroyl-hexoside	Yes	Yes
	O-Cinnamoyl O-coumaroyl O-galloyl-hexoside	Yes	Yes
	Trigalloyl derivative	-	Yes
	Phenylpropanyl heteroside derivative	-	Yes
Terpenes	Sesquiterpenes (general presence)	Yes	-
	Diterpenes (general presence)	Yes	Yes
	Triterpenes (general presence)	Yes	Yes

Compounds class	Compound name	Found in HGMO (***M. orbignyi***) [19]	Found in HGMQ (***M. q. anthidioides***) [12]
	Diterpenes ester (general presence)	Yes	-
Total Content (Quantified)	Total Phenolics	~118 mg GAE/g	118.7 ± 2.8 mg GAE/g
	Total Flavonoids	~25 mg QE/g	25.4 ± 2.8 mg QE/g

12. Santos, C.M.; Campos, J.F.; dos Santos, H.F.; Balestieri, J.B.P.; Silva, D.B.; de Picoli Souza, K.; Carollo, C.A.; Estevinho, L.M.; Santos, E.L.D., et al. Chemical composition and pharmacological effects of geopropolis produced by Melipona quadrifasciata anthidioides. Oxid. Med. Cell. Longev. 2017, 2017, 8320804. https://doi.org/10.1155/2017/8320804.

19. Santos, H.F.d.; Campos, J.F.; Santos, C.M.d.; Balestieri, J.B.P.; Silva, D.B.; Carollo, C.A.; De Picoli Souza, K.; Estevinho, L.M.; Dos Santos, E.L. Chemical profile and antioxidant, anti-inflammatory, antimutagenic and antimicrobial activities of geopropolis from the stingless bee Melipona orbignyi. Int. J. Mol. Sci. 2017, 18, 953. https://doi.org/10.3390/ijms18050953.
